# The pre-hospital 12-lead electrocardiogram is associated with longer delay and worse outcomes in patients presenting to emergency medical services with acute stroke: a linked cohort study

**DOI:** 10.29045/14784726.2022.09.7.2.16

**Published:** 2022-09-01

**Authors:** Scott Munro, Debbie Cooke, Mark Joy, Adam Smith, Kurtis Poole, Laurence Perciato, Janet Holah, Ottilia Speirs, Tom Quinn

**Affiliations:** University of Surrey; South East Coast Ambulance Service NHS Foundation Trust ORCID iD: https://orcid.org/0000-0002-0228-4102; University of Surrey ORCID iD: https://orcid.org/0000-0003-1944-7905; University of Oxford; Portsmouth Hospitals NHS Trust; Thames Valley Air Ambulance; South East Coast Ambulance Service NHS Foundation Trust; South East Coast Ambulance Service NHS Foundation Trust; Frimley Health NHS Foundation Trust; Kingston University and St George’s, University of London ORCID iD: https://orcid.org/0000-0002-5116-0034

**Keywords:** 12-lead electrocardiogram, emergency medical services, stroke

## Abstract

**Objectives:**

To investigate the association between pre-hospital 12-lead electrocardiogram (PHECG) use in patients presenting to emergency medical services (EMS) with acute stroke, and clinical outcomes and system delays.

**Methods:**

Multi-centre linked cohort study. Patients with verified acute stroke admitted to hospital via EMS were identified through routinely collected hospital data and linked to EMS clinical records via EMS unique identifiers. Ordinal and logistic regression analyses were undertaken to analyse the relationship between having a PHECG and modified Rankin Scale (mRS); hospital mortality; pre-hospital time intervals; door-to-scan and door-to-needle times; and rates of thrombolysis.

**Results:**

Of 1161 eligible patients admitted between 29 December 2013 and 30 January 2017, PHECG was performed in 558 (48%). PHECG was associated with an increase in mRS (adjusted odds ratio [aOR] 1.30, 95% confidence interval [CI] 1.01–1.66, p = 0.04) and hospital mortality (aOR 1.83, 95% CI 1.26–2.67, p = 0.002). There was no association between PHECG and administration of thrombolytic treatment (aOR 1.06, 95% CI 0.75–1.52, p = 0.73). Patients who had PHECG recorded spent longer under the care of EMS (median 49 vs 43 minutes, p = 0.006). No difference in times to receiving brain scan (median 28 with PHECG vs 29 minutes no PHECG, p = 0.32) or thrombolysis (median 46 vs 48 minutes, p = 0.37) were observed.

**Conclusion:**

The PHECG was associated with worse outcomes and longer delays in patients with acute ischaemic stroke.

## Background

Emergency medical services (EMS) play a crucial role in the assessment, management and transportation of acute stroke patients in the pre-hospital environment (Pulvers & Watson, 2017). EMS care in the United Kingdom incorporates stroke-recognition tools such as the face arm speech test (FAST) (Harbison et al., 2003) and national protocols to expedite transfer of patients to hospitals capable of providing thrombolysis or thrombectomy (Brown et al., 2019).

Due to the time-dependent nature of the disease and the available therapeutic options, focusing on pre-hospital interventions that are beneficial, while reducing delay to hospital care, is a priority for stroke systems. Studies have demonstrated that outcomes for patients depend on time to treatment, with lifetime benefits for each minute shorter stroke onset to receiving thrombolysis, emphasising the importance of constant improvement of treatment routines (Darehed et al., 2020; Fonarow et al., 2014; Meretoja et al., 2014). While decreases in door-to-needle (DTN) time have demonstrated improvements in patient outcomes, pre-hospital delays have shown little improvement in reducing onset-to-door time over the years (Evenson et al., 2009; Pulvers & Watson, 2017). The value of the pre-hospital 12-lead electrocardiogram (PHECG) in acute ischaemic stroke versus the time taken to undertake them is unknown. A systematic review did not identify any studies of PHECG use in stroke patients attended by EMS (Munro et al., 2018), and studies reported since 2015 have focused on PHECG use to capture arrhythmias and ambulance process times, respectively, rather than patient outcome (Bobinger et al., 2017; Drenck et al., 2019).

In the United Kingdom, all NHS EMS ambulances have PHECG capability. Paramedics are trained to record PHECG and interpret signs of acute myocardial infarction or ischaemia, and common arrhythmias. This, alongside availability of hospital data from the UK Sentinel Stroke National Audit Programme (SSNAP) (Bray et al., 2013), a prospective, longitudinal, multidisciplinary audit of stroke care for every acute hospital in England, Wales and Northern Ireland, provides an opportunity to study the use of PHECG in patients with acute ischaemic stroke.

The objective of this study was to describe the use of PHECG in EMS patients with acute ischaemic stroke and determine associations with patient outcomes and care processes. We hypothesised that there is a statistically significant difference in the modified Rankin Scale (mRS) between patients who had a PHECG recorded and those that did not.

## Methods

Favourable ethical opinion was gained from the NHS Research Ethics Committee Berkshire B (REC ref 16/SC/0528), and the UK Health Research Authority (HRA) approved the study.

### Design

We performed a multi-centre, linked cohort study, based on routinely collected data from participating hospitals submitted to SSNAP, and EMS patient clinical records (PCRs). PHECG was defined as any 12-lead electrocardiogram (ECG) recorded prior to arrival at hospital, while in the care of EMS. During the time of the data collection, national clinical guidelines for ambulance services in the United Kingdom recommended undertaking PHECGs in acute stroke patients, providing this did not result in prolonged pre-hospital time (Brown et al., 2016). There were no local guidelines on recording PHECG from the participating EMS.

The primary outcome for this study was mRS at discharge from hospital. Secondary outcomes included hospital mortality, EMS interval times, door-to-needle time and rates of thrombolysis received.

We conducted a multi-centre study, consisting of one large regional EMS serving a population of approximately 4.5 million and three district general hospitals providing hyper-acute stroke units (HASUs) in a mixed urban/rural county in the south of England. Thrombectomy services had not been introduced at the time of data collection.

Eligible patients were identified through hospital data collected as part of SSNAP between 29 December 2013 and 30 January 2017. SSNAP data do not include patients with subarachnoid haemorrhage, subdural and extradural haemorrhage or whose stroke was more than 28 days before presenting to hospital.

### Inclusion criteria

Patients were eligible for the study if they were recorded on the hospital SSNAP data as being confirmed ischaemic stroke or intracerebral haemorrhage and patients transported to hospital by the participating EMS. Patients diagnosed with transient ischaemic attack were excluded, as they are not recorded in SSNAP.

Hospital data were initially screened by the hospital clinical care team, where patient identifiable information was removed. The EMS unique identifier was used to link scanned paper copies of the PCRs completed by EMS staff.

EMS data were abstracted by three paramedics (KP, LP, AS), trained in EMS data abstraction, who adhered to explicit standardised protocols for case selection or exclusion in accordance with published guidance for chart reviews in emergency medicine (Kaji et al., 2014). A small pilot test was independently taken by one author (SM) to confirm the data collection procedures worked for the data source.

Standardised data extraction forms designed for this study were used to ensure uniform handling of data. One author (SM) performed a two-tailed z-test for transcription errors by the data abstractors on a randomly selected sample of EMS patient records. There was no evidence to reject the null hypothesis of a transcription error rate of 2%.

### Study variables

EMS time intervals, such as total scene time (time elapsed from arrival of EMS at scene and EMS leaving scene) and total EMS time (time elapsed from 999 call to EMS to patient arrival at hospital) were calculated. Door-to-scan (DTS) time was calculated from the amount of time elapsed from patient first arriving at hospital to time of first brain-imaging after stroke. DTN time was calculated from the amount of time elapsed from the patient first arriving at hospital to initiation of alteplase infusion. ECG findings were collected from a free-text box on the PCR where the EMS crews could write their interpretation of the recorded ECG.

#### Study size

A Mann-Whitney test was used to perform a power calculation to determine the required sample. Distribution data of mRS were taken from the Virtual International Stroke Trials Archive (VISTA) (Ali et al., 2007). 906 samples were required to power the study at 80% to detect a one-point shift in mRS. The level of significance was set *a priori* at 5%.

#### Statistical analysis

For descriptive statistics of baseline characteristics, percentages, means and standard deviations, as well as medians with the interquartile range (IQR), are reported.

Chi-square tests were used to test the difference in the categorical variables between patients in the PHECG and non-PHECG groups, and Kruskal-Wallis tests were used to compare continuous variables. The threshold for statistical significance was 0.05.

To analyse the relationship between having a PHECG and mRS, an ordinal regression was used, yielding a proportional odds ratio and 95% confidence intervals (CIs) (see Supplementary 1). During the statistical analysis, it was found that an mRS of 5 at discharge was reported less frequently, compared to other scores. Therefore, we banded mRS into three categories (0–2, 3–4, 5–6) and after testing we had no reason to reject the proportional odds assumption for the ordinal regression test. Logistic regression was used for dichotomous outcomes.

Our data contained 68 (5.85%) records with missing mRS scores and two (0.17%) records with missing ethnicity. We performed a sensitivity analysis by multiply imputing missing data (Van Buuren & Groothuis-Oudshoorn, 2011) and pooling estimates (using Rubin’s (2004) rules) of the coefficients of two logistic regression models obtained by dichotomising the discharge mRS score at 2 and 3.

We imputed 20 datasets, employing predictive mean matching, for each model. In both cases, the effect sizes of recorded PHECG remained significant (at the 5% level) and coefficient estimates were similar.

The statistical software package ‘R’ (version 3.3.1) was used to perform all statistical analyses (R Core Team, 2016).

## Results

**Figure 1:**
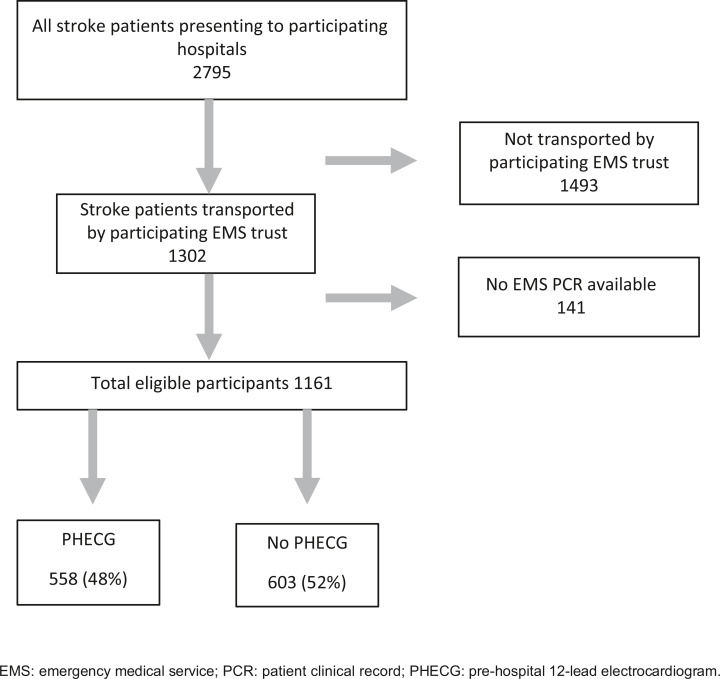
Figure 1. Participant flow chart.

Of 2795 patients admitted with confirmed ischaemic stroke from 29 December 2013 to 30 January 2017, 1493 (53%) were excluded as they were not transported by the participating EMS. EMS records were missing for a further 138 (5%). Therefore, 1161 (42%) patients were available for analysis (Figure 1).

PHECGs were performed in 558/1161 (48%) of patients (Table 1). Patients receiving PHECG were more frequently male (52% vs 46%, p = 0.05), and older (78 ± 13 vs 76 ± 14 years, p = 0.01) than those who did not. Hypertension was more common in patients who had no PHECG (52% vs 45%, p = 0.03). Patients with no stroke symptoms on arrival at hospital (National Institutes of Health stroke scale [NIHSS] 0) less frequently received PHECG (p = 0.005) than those with severe stroke symptoms (NIHSS 20–42) (p = 0.003).

**Table 1.** Baseline characteristics.

	Total = 1161	No PHECG (n = 603)	PHECG (n = 558)	p-value
Female sex	572 (49%)	314 (52%)	258 (46%)	0.05
Age, mean ± SD years	76 ± 13	76 ± 14	78 ± 13	0.01
				
White British	1115 (96%)	578 (96%)	537 (96%)	0.85
Mixed	2 (0.2%)	1 (0.2%)	1 (0.2%)	1
Asian or Asian British	17 (1%)	9 (1%)	8 (1%)	1
Other ethnic groups	25 (2%)	14 (2%)	11 (2%)	0.83
Not known	2 (0.2%)	1 (0.2%)	1 (0.2%)	1
				
Congestive heart failure	69 (6%)	35 (6%)	34 (6%)	0.93
Hypertension	564 (49%)	311 (52%)	252 (45%)	0.03
AF	213 (18%)	107 (18%)	106 (19%)	0.63
Diabetes	170 (15%)	91 (15%)	79 (14%)	0.71
Previous stroke/TIA	264 (23%)	138 (23%)	126 (23%)	0.96
Antiplatelets	67 (6%)	37 (6%)	30 (5%)	0.67
Anticoagulants	126 (11%)	65 (11%)	61 (11%)	1
				
No stroke symptoms (NIHSS 0)	153 (13%)	96 (15%)	57 (10%)	0.005
Minor stroke (NIHSS 1–4)	339 (29%)	178 (30%)	161 (29%)	0.85
Moderate stroke (NIHSS 5–15)	489 (42%)	246 (41%)	243 (44%)	0.37
Moderate to severe stroke (NIHSS 16–20)	94 (8%)	52 (9%)	42 (8%)	0.56
Severe stroke (NIHSS 20–42)	88 (8%)	31 (5%)	55 (10%)	0.003
				
First heart rate, bpm, median (IQR)	80 (23)	80 (21)	79 (28)	0.39
First systolic blood pressure, mmHg, median (IQR)	157 (42)	159 (40)	153 (43)	0.14
First diastolic blood pressure, mmHg, median (IQR)	86 (26)	87 (24)	86 (28)	0.53
				
FAST positive				
Yes	879 (76%)	454 (75%)	425 (76%)	0.78
No	222 (19%)	116 (19%)	106 (19%)	0.97
UTA	43 (4%)	20 (3%)	23 (4%)	0.56
Not recorded	16 (1%)	12 (2%)	4 (1%)	0.11

AF: atrial fibrillation; bpm: beats per minute; FAST: face arm speech test; IQR: interquartile range; NIHSS: National Institutes of Health stroke scale; PHECG: pre-hospital 12-lead electrocardiogram; SD: standard deviation; TIA: transient ischaemic attack; UTA: unable to attain.

PHECG was associated with an increase in mRS at discharge (aOR 1.30, 95% CI 1.01–1.66, p = 0.04) (Table 2). A total of 184 (16%) patients died in hospital during the study period. PHECG was associated with increased hospital mortality (aOR 1.83, 95% CI 1.26–2.67, p = 0.002). Of 189 (16%) patients receiving thrombolysis, 95 (50%) received a PHECG. PHECG was not found to be associated with receiving thrombolysis (aOR 1.06, 95% CI 0.75–1.52, p = 0.73).

**Table 2.** Association of pre-hospital 12-lead electrocardiogram on key clinical outcomes.

	aOR	95% CI	p-value
Discharge mRS	1.30	1.01–1.66	0.04
30-day hospital mortality	1.83	1.26–2.67	0.002
Thrombolysis use	1.06	0.75–1.52	0.73

aOR: adjusted odds ratio; CI: confidence interval; mRS: modified Rankin Scale.

Median time from EMS call to arrival at hospital was 5 minutes longer (60 minutes IQR 47–77) vs 55 minutes (IQR 43–71), p = 0.08) and the median time EMS personnel spent on scene before transportation to hospital was 7 minutes longer (35 (IQR 25–46) vs 28 minutes (IQR 21–39), p = 0.02) (Table 3).

**Table 3.** Time intervals.

	All (minutes)	PHECG (minutes)	No PHECG (minutes)	p-value
Time spent on scene, median (IQR)	32 (23–43)	35(25–46)	28 (21–39)	0.02
Total time under care of EMS, median (IQR)	45 (34–59)	49 (37–62)	43 (33–54)	0.006
EMS call to arrival at hospital, median (IQR)	58 (45–74)	60 (47–77)	55 (43–71)	0.07
DTS time, median (IQR)	29 (18–63)	28 (18–51)	29 (18–79)	0.32
DTN time, median (IQR)	47 (33–69)	46 (33–69)	48 (33–70)	0.37

DTN: door-to-needle; DTS: door-to-scan; EMS: emergency medical service; IQR: interquartile range; PHECG: pre-hospital 12-lead electrocardiogram.

There was no difference observed between the PHECG and non-PHECG patients in relation to DTN time for thrombolysis; median DTN 46 (IQR 33–69) versus 48 (IQR 33–70) minutes (p = 0.37). Having a PHECG recorded was not associated with DTS times; median DTS time 28 (IQR 18–51) versus 29 minutes (IQR 18–79) (p = 0.32).

Of the 558 patients who had a PHECG recorded, 363/558 (65%) had ECG abnormalities reported by EMS clinicians. The most frequently reported abnormality was atrial fibrillation (AF) (21.2%), followed by sinus tachycardia (6.8%), sinus bradycardia (5.2%) and right bundle branch block (4.7%). Of the participants, 9.7% had an ECG recorded, but no documented interpretation (Table 4).

**Table 4.** Emergency medical service-reported pre-hospital 12-lead electrocardiogram abnormalities.

EMS interpretation of PHECG	Total (n = 558)
Normal sinus rhythm	195 (35%)
AF	118 (21%)
ECG recorded but no interpretation noted	54 (10%)
Sinus tachycardia	38 (7%)
Sinus bradycardia	29 (5%)
Right bundle branch block	26 (5%)
Other	19 (3%)
Atrio-ventricular block	17 (3%)
Paced rhythm	16 (3%)
Ectopic beats / PVCs	16 (3%)
Left bundle branch block	11 (2%)
ST-elevation	6 (1%)
ST-depression	3 (0.5%)
Left ventricular hypertrophy	3 (0.5%)
T-wave inversion	3 (0.5%)
Ventricular tachycardia	2 (0.4%)
Non-specific T-wave abnormality	1 (0.2%)
QTc prolongation	1 (0.2%)

AF: atrial fibrillation; ECG: electrocardiogram; EMS: emergency medical service; PHECG: pre-hospital 12-lead electrocardiogram; PVC: premature ventricular contraction.

## Discussion

The results from this study suggest an association between PHECG use and worse functional outcome, higher hospital mortality and longer pre-hospital times in patients with acute stroke.

While our study found more deaths and worse functional outcomes in patients receiving PHECG, this could be explained by the patient groups that paramedics undertook PHECGs on. In this study, patients with a higher NIHSS on arrival at hospital were more likely to receive PHECG. It is likely that patients with a higher NIHSS presented to EMS as more unstable and required more EMS interventions on scene, or were more challenging to manage on scene, resulting in delayed transportation to hospital. While we attempted to adjust for NIHSS and other well-known risk factors in our regression models, it is likely that a larger sample would be required to be confident of an independent effect. Our team have recently explored the clinical decision-making surrounding EMS staff recording PHECGs in a qualitative interview study, which may provide further understanding as to the usefulness of the PHECG for acute stroke patients. We hope to publish the findings shortly.

The need to streamline pre-hospital processes for stroke patients is well recognised (Pulvers & Watson, 2017). Our findings that stroke patients who had PHECGs recorded had longer pre-hospital delays are similar to those of Drenck et al. (2019) who reported significantly shorter on-scene times for stroke patients when ECG was obtained in hospital as opposed to on scene (17 vs 21 minutes, estimated time reduction was 24%, RR = 0.76, 95% CI 0.64–0.90, p = 0.0015). Drenck et al. (2019) speculated that these delays may lead to negative outcomes for patients. Results from our study confirm an association between PHECG and worse functional outcome. Li et al. (2021) recently reported that on-scene time comprised 38.5% of total pre-hospital time in acute stroke, and recommended evaluation of procedures such as PHECG being undertaken during transport to hospital rather than delaying on scene.

Previous studies have reported that PHECG monitoring is a useful tool in detecting rates of abnormalities in stroke patients and suggest that the detection of cardiac arrhythmia in the pre-hospital setting may add to the hospital evaluation of patients. Bobinger et al. (2017) report that AF was captured on PHECG in 7% (18/259) of patients, and in 0.7% (2/259) AF was only seen on PHECG and did not reoccur on hospital admission. The low rate of AF detected compared to our study could potentially be due to the difference in sample sizes and population studied as well as the interpretation of PHECG by non-physician EMS personnel in our study, while Bobinger et al. (2017) used experienced investigators following parameters according to published guidelines (O’Keefe et al., 2008). Whether the fact that ECG abnormalities were reported in 65% of our patients would have changed management or outcome remains moot.

Lyckhage et al. (2020) compared the rates of detecting AF between pre-hospital continuous ECG (cECG) monitoring and in-hospital 12-lead ECG and found no difference in the probability of being diagnosed with AF (pre-hospital cECG 5.4% (95% CI 3.6–7.8) vs in-hospital 12-lead ECG 6.1% (95% CI 4.1–8.7), p = 0.68).

Our study has several limitations. Firstly, the observational design precludes establishing a causal relationship between PHECG and the reported outcomes. While an association was reported in the statistical analysis, this may be due to other factors, such as EMS decision-making on undertaking PHECG on patients with more severe stroke symptoms. Secondly, our analysis was dependent on the extent and validity of data recorded for SSNAP. We were unable to access longer-term outcomes, such as mRS at 6 months, as has been suggested in other studies (Lees et al., 2012). Measuring mRS at the point of discharge may not consider the potential continued degree of recovery following discharge from hospital (Lees et al., 2012). Moreover, while the proportion of patients using EMS appeared low (45%), this could be explained by use of transportation to hospital by non-participating EMS in neighbouring regions. Thirdly, while we had access to the attending EMS interpretation of the ECG, we were unable to access copies of ECGs to facilitate independent interpretation. Finally, our focus was solely on patients with confirmed stroke. Given the nature of the SSNAP data, we were unable to explore patients in whom EMS suspected stroke but did not have the diagnosis subsequently confirmed.

Strengths of this study include its multi-centre nature. In addition, robust measures were put in place to increase the accuracy and consistency of data collection. To our knowledge, this is the first study to report the association of recording a PHECG by EMS clinicians in acute stroke patients with the investigated outcomes. As such, our findings represent the best available evidence on the use and impact of PHECGs in stroke patients. Larger, prospective studies with longer follow-up periods are required to confirm or refute our findings.

## Conclusion

The PHECG was associated with worse outcomes and longer delays in patients presenting to EMS with acute ischaemic stroke; however, this may be due to EMS decision-making on undertaking PHECG on patients with more severe stroke symptoms. Further exploration of EMS decision-making on the use of PHECG is recommended.

## Author contributions

SM: Designed and conceptualised study; analysed data; drafted manuscript for intellectual content. DC: Designed and conceptualised study; interpreted data; revised manuscript for intellectual content. MJ: Designed study; analysed data; interpreted data; revised manuscript for intellectual content. AS: Major role in acquisition of data; revised manuscript for intellectual content. KP: Major role in acquisition of data; revised manuscript for intellectual content. LP: Major role in acquisition of data; revised manuscript for intellectual content. JH: Lay representative; designed study; revised manuscript for intellectual content. OS: Major role in acquisition of data; revised manuscript for intellectual content. TQ: Designed and conceptualised study; interpreted data; revised manuscript for intellectual content. All authors read and approved the final manuscript. TQ acts as the guarantor for this article.

## Conflict of interest

TQ reports grants from the National Institutes of Health Research and British Heart Foundation. All other authors declare no conflict of interest.

## Ethics

Favourable ethical opinion was gained from the NHS Research Ethics Committee Berkshire B (REC ref 16/SC/0528), and the UK HRA approved the study. As this was a secondary analysis of routinely collected, non-identifiable data, patient consent was not required.

## Funding

This work was supported by a School of Health Sciences PhD bursary – University of Surrey, and the South East Coast Ambulance Service NHS Foundation Trust.

## Data availability statement

Participating organisations withheld permission for data sharing.
